# Tyrosine kinase inhibitor associated vascular toxicity in chronic myeloid leukemia

**DOI:** 10.1186/s40959-015-0008-5

**Published:** 2015-12-04

**Authors:** Oren Pasvolsky, Avi Leader, Zaza Iakobishvili, Yishay Wasserstrum, Ran Kornowski, Pia Raanani

**Affiliations:** 1 Hematology Institute, Davidoff Cancer Center, Beilinson Hospital, Rabin Medical Center, 39 Jabotinsky Street, Petah Tikva, 49100 Israel; 2 Department of Cardiology, Beilinson Hospital, Rabin Medical Center, Petah Tikva, Israel; 3 Sackler School of Medicine, Tel Aviv University, Tel Aviv, Israel

**Keywords:** Vascular adverse events, Chronic myeloid leukemia, Nilotinib, Platelets, Ponatinib, Tyrosine kinase inhibitor

## Abstract

Tyrosine kinase inhibitors (TKIs) have revolutionized the management and outcomes of chronic myeloid leukemia (CML) patients. Improved disease control and prolonged life expectancy now mandate focus on improving TKIs’ safety profile. Recently, vascular adverse events (VAEs) have emerged as a serious consequence of some of the newer TKIs. In this review, we describe the clinical spectrum of TKI-associated VAE, and examine the unique vascular safety profile of the main TKIs currently used in the treatment of CML: imatinib, nilotinib, dasatinib, bosutinib and ponatinib. The issue of TKI-related platelet dysfunction is discussed as well. We describe the contemporary research findings regarding the possible pathogenesis of the VAE. Finally, the different aspects of TKI-associated VAE management are addressed, including prevention methods, monitoring strategies and treatment options.

## Background

Over a decade ago, the International Randomized Interferon vs. STI571 Study (IRIS) established the tyrosine kinase inhibitor (TKI) imatinib as the standard of care for chronic myeloid leukemia (CML) patients [1]. Thus, imatinib revolutionized the treatment and prognosis of CML, which transitioned from a disease which progressed to acute leukemia and death within a matter of years, to a truly chronic disease with an 85 % eight year overall survival (and even 93 % overall survival taking into consideration only CML related death), close to that of the general population. During the following years the emergence of newer generations of TKIs has presented CML patients with several highly effective therapeutic options. Each TKI has a unique side-effect profile, which together with the disease and patient characteristics help determine the choice of TKI in a given patient. Recently, TKI-associated vascular adverse events (VAE), seen with some of the TKIs, have emerged as one of the most perturbing and poorly understood TKI side effects. Consequently this has become one of the main challenges in the contemporary management of CML. This review focuses upon the clinical spectrum of TKI-associated VAE, its proposed pathogenesis and the unique vascular safety profile of the main TKIs currently used in the treatment of CML: imatinib, nilotinib, dasatinib, bosutinib and ponatinib. Finally, we propose an approach to prevention and management of VAEs in this patient population. Hypertension is a known cardiovascular complication of TKIs, comprehensively reviewed elsewhere in this issue by our group. This manuscript will focus on discussion of the vascular effects of the TKIs.

## The use of TKIs in CML

In the vast majority of cases, CML arises due to the reciprocal translocation between chromosomes 9 and 22, carrying the ABL1 and BCR genes respectively, resulting in a truncated chromosome 22, known as the Philadelphia chromosome. The resulting chimeric *BCR*-*ABL* gene produces a constitutively active tyrosine kinase [2]. TKIs block the binding of adenosine triphosphate (ATP) to the BCR-ABL1 tyrosine kinase, thus inhibiting its activity. The pivotal phase III IRIS study comparing first line imatinib to the previous standard of care (interferon alpha and cytarabine) showed an unprecedented complete cytogenetic response rate of almost 86 % in chronic phase CML. Nevertheless, some of these patients later relapsed due to the development of resistance to the drug. This translated into overall survival similar to that of the general population, thus drastically improving the dismal long-term outlook for CP-CML patients. The cornerstone of response assessment is the cytogenetic and molecular response. The achievement of treatment milestones is based on these variables at different time points [3], and includes the definition of treatment failure, optimal response and warning signs for each time-point.

Nilotinib and dasatinib are 2^nd^ generation TKIs, and have a greater affinity to the binding site on the BCR-ABL1 tyrosine kinase protein compared to imatinib, allowing for more effective BCR-ABL1 inhibition [4]. Both drugs have demonstrated greater response rates compared to imatinib as first-line treatment for chronic phase CML in phase III trials [5, 6], and are considered as options for 1^st^ line treatment in chronic phase CML. Bosutinib, another 2^nd^ generation TKI, has not yet shown response rate superiority compared to imatinib [7], and thus is indicated for patients with resistance or intolerance to previous therapy [3]. Ponatinib, a 3^rd^ generation TKI, is potent against resistant cases of CML including those carrying the T315I mutation [8].

Mutations in the BCR-ABL1 kinase domain are one of the key mechanisms responsible for TKI resistance, and hence different mutations have been shown to confer different sensitivities of the various TKIs. In current practice, mutational analysis of *BCR*-*ABL1* is performed only if the patient fails to respond to first line treatment or beyond. The T315I mutation is insensitive to all TKIs in clinical use, except for ponatinib. With the addition of more TKIs to the armamentarium of treatment in CML, focus has shifted towards the management of adverse effects, improvement of adherence, treatment discontinuation, management of drug resistance, and ultimately personalizing treatment according to disease characteristics and patient’s comorbidities.

## Clinical spectrum of TKI-associated vascular events

Over the past few years evidence of varying quality has emerged demonstrating an association between treatment with certain TKIs and vascular toxicity [9]. It is important to note that the vascular safety issues with TKI treatment in CML were initially unnoticed. However, as clinical experience with the newer TKIs (namely - nilotinib, dasatinib, bosutinib and ponatinib) evolved, it became clear that further investigation regarding this issue is warranted. The main VAEs recognized thus far have been peripheral arterial occlusive disease (PAOD), although others include cerebral ischemia, myocardial infarction and pulmonary hypertension. Although nomenclature guidelines have aimed to achieve unity in the field of vascular disease in general, reported events in clinical trials may not be fully compliant with such guidelines, causing data heterogeneity. The 2008 American Heart Association (AHA) guidelines for nomenclature of vascular disease [10] propose the term peripheral artery disease (previously PAOD) to describe all upper and lower arterial events, acute or chronic, excluding renal, coronary, cerebral, mesenteric disease. Venous events have also been reported, especially with ponatinib. Some rare cases of arterial vasospastic phenomena, such as Reynaud syndrome, have been described after nilotinib use as well [11, 12]. The closely related issue of TKI associated platelet dysfunction will also be addressed separately in this review. Most reports regarding VAE have been retrospective in nature, mostly pertaining to nilotinib, though data regarding ponatinib and other TKIs is now starting to be unveiled as well. Both retrospective and emerging prospective data on VAE almost exclusively examine vascular toxicity while under TKI therapy, and not before and after therapy, barring several case-reports. The overall incidence of events varies considerably between reports, depending on the TKI studied, the definition of VAE, the patient population and method of data collection (i.e. whether clinical disease or imaging studies were reported). It has become clear, however, that the different TKIs have different vascular safety profiles (Table 1) [13].

**Table 1:** Distribution of TKI-associated VAEs and other relevant toxicities, among the various TKIs in CML

	Imatinib	Nilotinib	Dasatinib	Bosutinib	Ponatinib
PAOD		++		+/−	++
IHD/CVA		+			+
VTE					+
Pulmonary hypertension			+		
Platelet dysfunction			+		+
Hypertension				+	++
Hyperglycemia	^a^	+			
Dyslipidemia	^a^	+			

*CVA* cerebrovascular accident, *IHD* ischemic heart disease, *PAOD* peripheral arterial occlusive disease, *TKI* tyrosine kinase inhibitor, *VAE* vascular adverse event, *VTE* venous thrombo-embolism

^a^Imatinib has been shown to have positive effects on glucose blood levels, as well as lipid profile (See text for details)

### Imatinib

Imatinib, the veteran of the TKIs, has been in routine clinical use as first line therapy since 2001. It is widely accepted that imatinib does not cause significant long term VAE, and many reports pertaining to the other TKIs’ safety profile have been in comparison to the “innocent” profile of imatinib, as discussed below. Furthermore, it has been suggested that imatinib may actually have beneficial vascular effects, leading to a reduced risk of VAEs. A retrospective analysis of three phase III trials with different TKIs [14] divided the participants of those trials into three groups: patients who received imatinib, patients who received nilotinib and those who received no TKI. The analysis revealed decreased rates of PAOD in the imatinib treated patients as compared to patients who were not treated with a TKI, and nilotinib treated patients had similar rates of PAOD as the no TKI group. The positive vascular effect of imatinib can also be suggested by positive effect of the drug in patients with pulmonary hypertension [15]. The above data should also be taken together with reports of improved lipid profiles and lowering of blood glucose levels under imatinib treatment [16, 17]. In summary, imatinib has a safe vascular profile and the above data show that caution and an understanding of the literature is needed when using imatinib as a control group in a comparison of VAEs with another TKI.

### Nilotinib

Of the newer generation of TKIs, the most reports regarding possible VAEs have been about nilotinib. One of the first reports of TKI associated VAEs came in 2011, when Aichberger et al. [18] published a report describing the results of a follow-up of 24 consecutive patients receiving nilotinib for CML. Using clinical examination, ankle-brachial pressure index (ABI) and duplex sonography of major arteries to screen for peripheral arterial occlusive disease (PAOD), three patients were observed to have a rapidly progressive and treatment-resistant severe arterial occlusive disease. In two of the patients the PAOD developed within a year of initiating nilotinib, and in all three patients the PAOD involved small vessels of the lower extremities, a distal form of PAOD resembling the PAOD found in diabetic patients. Further reports strengthened the notion that such VAEs can arise in some patients rapidly after initiation of TKI treatment. One such report was published by Hadzijusufovic et al. [19], who reported the clinical outcomes of 34 patients receiving nilotinib, showing an increased percentage of patients suffering from arterial occlusive disease (AOD) and severe AOD compared with several matched control groups (26.5 and 17.6 %, respectively) after a median observation time of 24 months. Another such report by Coon et al. [20] described a patient on nilotinib treatment with rapidly progressive intra- and extra-cranial atherosclerosis leading to stroke. The magnitude of the problem can be seen in the report by Kim et al. [21], who prospectively screened 159 patients treated for chronic-phase (CP)-CML with either imatinib or nilotinib for PAOD, using ABI and duplex ultrasonography. Pathological ABI was more prevalent in patients treated with second- or first-line nilotinib (35.7 and 26 %, respectively) compared to patients on first-line imatinib (6.3 %), corresponding with a remarkable 10.3 relative risk when comparing first-line nilotinib to first-line imatinib treatment. Only patients with current or previous treatment with nilotinib had clinically overt PAOD (5 patients). Another retrospective comparison between second line treatment with either nilotinib or dasatinib has shown an excess of vascular events with nilotinib. During a median observation time of 28 months, 11 % of nilotinib treated patients developed adverse vascular events, compared to 4 % in the dasatinib treated group [12].

The main auxillary test used in identifying subclinical occlusive arterial disease has been the ankle brachial index (ABI). A recent study demonstrated subclinical sonographic findings in 53 % of patients treated with nilotinib, whereas other parameters of vascular function, such as intima media thickness were not significantly different from healthy controls [22]. Data collected thus far has provided a wide range of VAEs associated with nilotinib treatment, mostly PAOD. The initial publication by Saglio et al. comparing nilotinib with imatinib as first line treatment for CML did not provide data regarding VAE [5]. However, the 3-year follow up of ENESTnd [23] revealed 7 patients out of the 556 patients (1.3 %) in the nilotinib group who developed PAOD. Thus, the incidence of nilotinib induced VAE has been reported in a considerably wide range, usually between 1 and 25 % [19, 23]. This is probably due to, in part, differences in research methodology, surveillance duration and patient selection. Reports that included overt clinically evident VAE have usually described a lower risk, such as the 2 % (5 out of 233 nilotinib treated patients) reported by Quintás-Cardama [11], whereas occult radiological findings compatible with atherosclerotic plaques have been reported in up to 53 % [22]. The rate of venous VAE does not seem to be increased with nilotinib treatment [23]. In summary, VAEs, especially arterial, are certainly a phenomenon that requires early and long-term monitoring, vigilance, and perhaps appropriate patient selection, when prescribing nilotinib, as discussed later in this review.

### Dasatinib

There is much less data regarding possible vascular-related safety issues with dasatinib. However, this drug’s unique vascular profile includes an uncommon but potentially life-threatening side-effect of pulmonary arterial hypertension (PAH), while most other VAEs are rare and probably do not occur at a higher frequency compared with the general population. In an analysis of 11 clinical trials involving dasatinib treatment for CML, the incidence of PAOD was low, 0.2 % out of a total of 2705 patients. All patients who developed PAOD were noted to have risk factors for the disease [24]. Dasatinib use has been associated with an increased risk of PAH. Original data from the DASISION trial, comparing dasatinib and imatinib frontline treatment of CML, did not reveal any cases of PAH [6]. However, a 3 year follow up of this trial [25] revealed 8 cases of PAH in the dasatinib arm, with no such cases in the imatinib arm. In this study, PAH diagnosis relied on echocardiography, while only one patient underwent right heart catheterization, which did not confirm the diagnosis. Several other case studies have strengthened this concern [26, 27], with the most informative report emerging from analysis of the French pulmonary hypertension registry. The analysis revealed 9 patients who developed PAH after treatment with dasatinib. Most of these patients had severe symptoms and marked hemodynamic instability, and both clinical and hemodynamic improvements usually occurred after dasatinib discontinuation, without the need for specific PAH-targeted therapy. There were no reported cases of PAH in patients with CML treated with other TKIs [28]. In summary, PAH is the only VAE associated with dasatinib. It is a rare, unique to this drug, and (at least in some cases) potentially resolves spontaneously after discontinuing dasatinib treatment.

### Bosutinib

A recent meta-analysis comparing TKIs and reference therapy (i.e. imatinib) showed no increased vascular toxicity with bosutinib [29], similar to recently published real-life data [30]. It should be kept in mind, though, that bosutinib inhibits some of the pathways inhibited by ponatinib, such as the SRC family kinases. A recently published meta-analysis [31] has in fact shown increased pooled incidence of cardiovascular events with bosutinib compared with imatinib and dasatinib. However, this metanalaysis was performed on non-randomized data, with only one study included that evaluated bosutinib. In summary, the contemporary data is scarce but is yet to reveal worrying signals of vascular toxicity. Nevertheless, as the use of this drug becomes more common outside of clinical trials, and until the pathogenesis of the vascular events associated with the above drugs is fully understood, real-life post-marketing data on vascular events in bosutinib-treated patients should be closely monitored.

### Ponatinib

Ponatinib, being the treatment of choice for patients with the T315I mutation, has been another focus of worrisome reports regarding an increased incidence of VAEs. Nicolini et al. [32] published a report of a prospective analysis of 19 patients who received treatment with ponatinib. Forty-two percent of the patients had arterial cardiovascular events after a median period of 8.5 months (range 4–17 months). A follow-up report on a phase I study of ponatinib has shown that there was a significant percentage of vascular occlusive events after a median follow-up of 33 months [33]. There were VAEs in 37 % of study subjects, of which 23 % were serious events, including (in descending order of frequency) cardiovascular, peripheral vascular, cerebrovascular and venous thrombotic events. In this report venous VAE occurred in 5 % of patients, none of which were considered to be serious events. Manufacturer prescribing information [34] reveals an alarming rate of both arterial and venous VAE of 27 % in phase I and II studies, as recently reviewed [35]. Arterial occlusive disease occurred in 20 % of patients, and venous thromboembolic events, mostly deep vein thrombosis and pulmonary embolism, occurred in 5 % of patients. In a recent phase II trial examining ponatinib as first line treatment for chronic phase CML, hypertension (new onset or worsening) was observed in an alarming 29 % of patients. Of note, hypertension was well controlled and reversible after ponatinib discontinuation or dose reduction [36]. In summary, although limited data has been published, ponatinib seems to be associated with a substantial risk of VAE, mainly arterial, but also venous. Hypertension is a common adverse effect and warrants attention and monitoring, but detailed discussion of this issue is beyond the scope of this review.

## Platelet dysfunction

Although most attention concerning vascular safety of TKIs has been focused on their pro-thrombotic effects, it should be noted that dasatinib and ponatinib have been associated with platelet dysfunction, potentially raising the risk for bleeding. This is also of interest as platelets are involved in the pathogenesis of vascular disease, in general. Both dasatinib and ponatinib have been shown to induce platelet dysfunction, as measured by PFA-100, a sensitive measure of primary hemostasis [37, 38]. The exact mechanism of this TKI induced platelet dysfunction has yet to be fully explained, although some research has shown that SRC family kinases (SFKs) inhibition, or interaction with some of the downstream components of the Src pathway in platelets by some of the TKIs, can cause decreased platelet function both in vitro and in vivo [39, 40]. Furthermore, as with other TKIs, mild thrombocytopenia may occur with the use of dasatinib, which has been shown to impair megakaryocyte formation [41]. The clinical impact of these findings needs further research as well. In a combined report of 4 multicenter studies with dasatinib, up to 10 % of patients experienced grade 3 or 4 bleeding events [42], while a study by Nazha et al. [38] found that ponatinib did not alter the rate of clinically significant bleeding. In addition, the effects of TKIs on platelet number and function may be relevant especially when anticoagulation and anti-platelet drugs are indicated in ponatinib and dasatinib-treated patients with established vascular disease.

### Off-target activity of TKIs

**Fig. 1:**
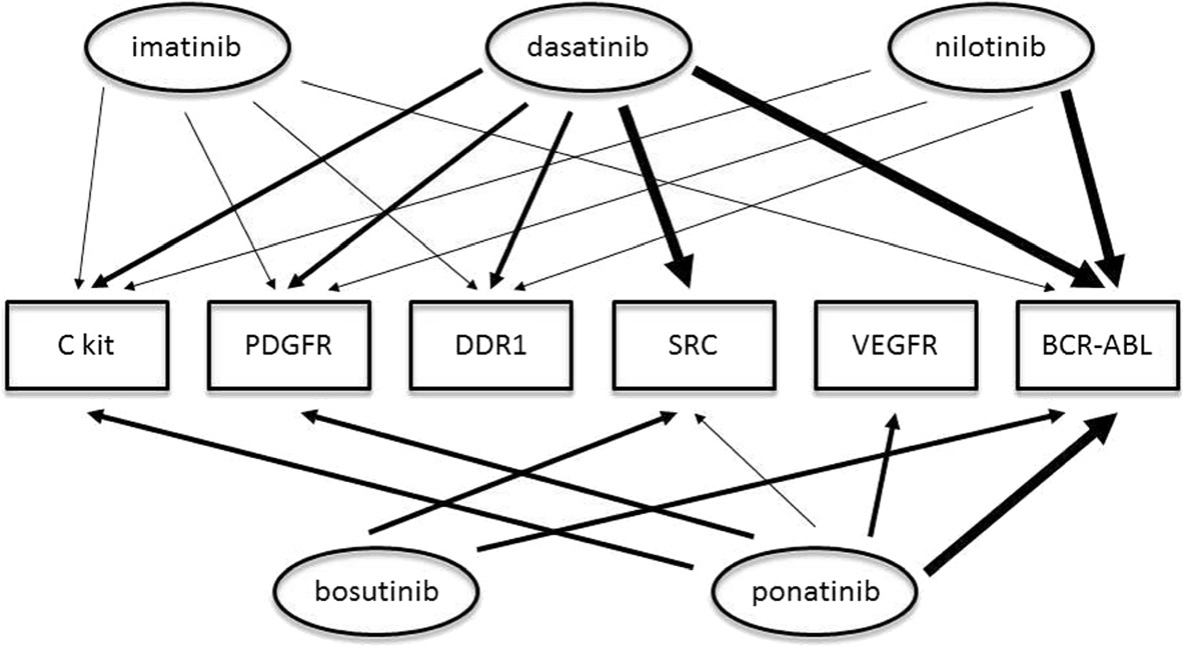
Comparative potency of BCR-ABL1 inhibition and selected off-target activity of tyrosine kinase inhibitors (TKIs). The figure describes the relative potencies of the various TKIs for BCR-ABL inhibition, as well as selected off-target activity of the various TKIs. Arrow width indicates potency. See text for further discussion

Alongside the therapeutic activity of the various TKIs on the BCR-ABL1 kinase target, each drug in this class has a unique off-target activity profile (Fig. 1). This selectivity may provide clues to the wide array of adverse effects encountered in clinical practice in TKI-treated CML patients. The works of Bantscheff et al. [43] and Rix et al. [13] revealed a detailed analysis of these non-kinase targets, providing the grounds for theoretical explanations for the selective vascular toxicity profile of the TKIs. For example, DDR1 is believed to play a role in atherosclerosis, and is mainly targeted by dasatinib and nilotinib, and perhaps by imatinib as well. Targeting of NQO2 by imatinib and nilotinib may lead to unwanted drug-drug interactions. PDGF-R and C-kit could potentially provide explanations for the vascular toxicity since they both effect the regulation of vascular and perivascular cells [13], however they are targeted by many of the TKIs, possibly excluding bosutinib [44].

The hypertensive effect of ponatinib is likely mediated by its off-target inhibition of VEGF-R. Indeed, other TKIs that target VEGF-R such as sorafenib, used for other malignancies, have been shown to cause hypertension in up to 19 % of patients [45].

Potent inhibition of SRC family kinases by dasatinib may explain its inhibitory effect on cell growth in both normal and neoplastic cells, as well as its potential to cause centrosomal aberrations and decrease in mitotic spindles [46].

It is unclear whether selectivity data alone will be able to explain why only some TKIs have the potential for vascular toxicity. For example, DDR-1, implicated in the formation of the atherosclerotic plaque, is recognized by both nilotinib and imatinib, whereas the clinical vascular phenotype is vastly different between these two TKIs, as described above. It may be that DDR1 has distinct effects in different situations, since inhibition of DDR1 has been shown to promote but also decrease atherosclerosis [47, 48]. Of note, nilotinib seems to have a particularly high inhibitory potency for DDR-1, higher than its effect on BCR-ABL1 [49]. Thus, it is possible that differential molecular targeting by the different TKIs play an important role in a more intricate pathophysiological process leading up to clinical vascular toxicity. However, our understanding of the interplay between these various off-target effects is in its infancy. Therefore, at the moment, knowledge of off-target effects is mainly useful in driving preclinical studies, possibly in selection of more suitable future TKIs, and guiding vigilance and preemptive monitoring in clinical trials.

## Pathogenesis of VAE

The mechanism of the increased tendency for vascular events with the newer TKIs remains poorly understood, currently relying on limited clinical and laboratory data. The clinical and in vitro studies indicate that perhaps a form of accelerated atherosclerosis could explain at least some of the vascular events seen in patients treated with TKIs. Nilotinib is associated with several metabolic disturbances, including hyperglycemia, perhaps via insulin resistance [50], and dyslipidemia which may develop within as little as three months of treatment [51]. However, these metabolic disturbances certainly do not explain all cases of VAE [52], and probably represent one of the multiple contributory mechanisms. Hypertension has also been reported in 9 % of patients treated with ponatinib in a phase II trial [53], and to a lesser extent, with bosutinib [54]. Manufacturer prescribing information data for ponatinib reveal much higher rates of hypertension (67 %), with up to 2 % of patients in clinical trials experiencing emergent symptomatic hypertension [34].

Hadzijusufovic et al. [19] explored the pathogenesis of nilotinib-associated VAEs in a multi-faceted laboratory model: First, in vitro effects were studied, using cultured human umbilical vein endothelial cells (HUVEC), human coronary artery –derived endothelial cells (HCAEC) and the human microvascular endothelial cell line HMEC-1. Nilotinib was found to inhibit the proliferation of endothelial cells, inhibit migration of HUVEC in a wound-scratch assay and also inhibit angiogenesis in a tube-formation assay. Second, in a mouse model of hind limb ischemia, nilotinib was found to slow blood flow-recovery after induction of ischemia, which was accompanied by increased rate of limb necrosis. These effects were not seen in the imatinib treated mice, which also exhibited greater microvessel density compared to the nilotinib treated mice. Furthermore, nilotinib was shown to promote the expression of pro-atherogenic cytoadhesion molecules (CAM) on HUVEC, including ICAM-1 (CD54), VCAM-1 (CD106) and E-Selectin (CD62E). Using chemical proteomics profiling and phosphor-array analysis, nilotinib, and not imatinib, was shown to bind to several antigenic targets in endothelial cells, including Tie-2/TEK, JAK1, BRAF and EPHB2. Both nilotinib and imatinib induced significant depletion of KIT+ mast cells, and both did not exhibit effects on platelet adhesion or aggregation, as tested in vitro and in vivo.

Katgi et al. [55] further tested the effects of nilotinib on function and viability of human carotid artery endothelial cells (HCtAECs). Nilotinib reduced proliferation of the HCtAECs, in a mild, albeit statistically significant manner, with dose-dependence. Next, levels of nitric oxide, von Willebrand factor, tissue plasminogen activator, plasminogen activator inhibitor 1, and endothelin 1 were evaluated. Secretory functions of the HCtAECs, both prothrombotic and anti-thrombotic, were not significantly affected by nilotinib.

Aprile et al. [56] reported on 75 patients who received either imatinib (*N* = 39) or nilotinib (*N* = 36) and were all evaluated during a routine visit for classical cardiovascular risk factors (such as diabetes mellitus and cigarette smoking) and were screened for PAOD and other atherothrombotic events. In addition, the following additional blood tests were performed: sCD40L level and endogenous thrombin potential (ETP) which are markers for platelet and coagulation activation; oxidized LDL (oxLDL) which is an early stage atherogenesis promoter; IL6, IL10, TNF-alpha which give an indication of the pro/anti-inflammatory balance; 3′UTR polymorphism of OLR1, which encodes for the oxidized LDL receptor 1 (LOX-1), providing information regarding the genetic predisposition for atherothrombotic events. Twenty-five percent of the patients receiving nilotinib developed PAOD, acute coronary syndrome or cerebral ischemia, as compared to 7.6 % of the patients receiving imatinib. Nilotinib treated patients had an unbalanced pro/anti-inflammatory network. The authors hypothesized that this pro-inflammatory state could cause pro-atherothrombotic activation via enhanced lipid peroxidation, and that genetic pro-atherothrombotic predisposition conferred by LOX-1 may play a role in the increased incidence of vascular events. Our group has recently used a HUVEC-based tube formation assay to demonstrate that ponatinib exerts a suppressive effect on neo-angiogenesis of vascular endothelial cells [57]. To date, there are no published laboratory data on the pathogenesis of ponatinib or dasatinib associated VAEs.

## Management

### Prevention and risk assessment

Treatment of CML patients is undergoing a fundamental paradigm shift. It is clear that all the above drugs, despite the vascular events of some, have a place in the contemporary armamentarium for treating CML. The key lies in selecting the patients at a minimal risk for such events, bearing in mind that each drug has its own side effect profile. Therefore, understanding the risk-benefit ratio for a given patient is crucial. However, it has yet to be proven whether TKI-associated VAE can be prevented by improved patient selection. One approach being investigated is stratification according to cardiovascular risk scores. This leans upon the hypothesis that atherosclerosis plays a key role in the pathogenesis of TKI-induced VAEs and that those patients with pre-existing atherosclerosis and conventional risk factors thereof, are consequently at a greater risk of TKI-induced VAEs.

Rea et al. [58] reported risk-stratified data of artery occlusive disease (AOE) in 75 CML patients treated with nilotinib, using the European Society of Cardiology (ESC) 2012 classification. Seventy-two percent of patients in the high/very high CVD risk group had AOE at 48 months, as opposed to 12.13 % in the low/moderate CVD risk group. Further validation of risk stratification for potential nilotinib prescription according to the Systemic Coronary Risk Evaluation Project (SCORE) chart as part of the 2012 ESC classification has been shown in other retrospective analyses [59, 60]. Of all the prevention strategies, risk-adapted selection of patients carries the most promise and will improve once the pathogenesis of these VAEs is better understood.

Although the 2013 ELN guidelines [3] briefly mention the importance of the comorbidity profile in drug-selection, a recent review of current concepts in TKI selection puts a greater emphasis on this issue [61]. However, in some circumstances, drugs with potential vascular toxicity may be essential for a particular individual. Such may be the case for a patient with cardiovascular risk factors in need of ponatinib due to CML with the T315I mutation. Although evidence in this area is still lacking, it seems reasonable to try and reduce potential ponatinib VAE by improved comorbidity control. Perhaps preemptive treatment with certain drugs which target the hypothetical pathway of VAE pathogenesis in these patients, such as aspirin and statins, may decrease the chances of developing VAE or reduce their severity. However, this strategy is theoretical and yet to be proven, and warrants further research, especially in the case of aspirin or anticoagulant treatment in patients receiving ponatinib and dasatinib which cause platelet dysfunction [37, 38]. In general, it makes sense to ensure that risk factors for atherosclerosis are at least managed according to contemporary guidelines for the general population [62], especially in patients receiving ponatinib and nilotinib.

It may be prudent to perform an echocardiogram prior to starting treatment with dasatinib, since this drug has been shown to cause PAH. This may identify patients with raised basal pulmonary arterial pressures, perhaps at increased risk for this complication. It is not clear whether this places patients at higher risk for this uncommon side effect, but could dictate a different monitoring strategy.

### Monitoring for VAEs

Until further data is collected, it seems reasonable to recommend periodic monitoring of known metabolic changes that can be inflicted by certain TKIs. Cardiovascular risk scores [60] may guide us to which patients should be monitored more intensely. Patients prescribed TKIs should have periodic lipid profiling and blood glucose examinations, as well as blood pressure monitoring. Patients receiving nilotinib, ponatinib and to a lesser extent, dasatinib, should be aware of the subtle symptoms of relevant VAEs and the treating physician should actively search for such symptoms in the history and physical examination. An active field of current research is subclinical markers of vascular toxicity, which may be used to detect early signs of vascular disease, which could change management. In a small prospective study (*N* = 15), a significantly higher rate of atherosclerotic plaques was found in nilotinib treated patients (53 %) compared with healthy controls (13 %). A matched group of patients with metabolic syndrome had plaque rates similar to those treated with nilotinib. Other signs of subclinical atherosclerotic disease, such as intimal media thickness (IMT) and ABI were not significantly different between the groups [22]. Some centers already routinely use ABI to monitor nilotinib and ponatinib patients, however this strategy has not been evaluated prospectively, and questions remain regarding subclinical thresholds for changing TKI treatment or dose and managing atherosclerosis. It is clear that this is a promising field for future research, as this may allow earlier interventions to prevent overt clinical VAE.

### Treatment

The relevant specialist, such as vascular surgeons, cardiologists or neurologists, should initially treat any VAE like any non-TKI associated vascular event. The hematologist should naturally be the case manager of this patient as drug interactions with TKIs, other relevant TKI-associated adverse effects and decisions regarding withholding and/or switching treatment are all on the cards. From a hematological viewpoint, the major decision is whether to discontinue the suspected offending TKI and switch to a different drug, which depends upon the severity of the vascular event on the one hand, and the response state of the CML and other alternative treatment options for CML, on the other. Discontinuation seems sensible in the case of severe vascular events, such as a myocardial infarction or cerebrovascular accident, as well as severe PAOD. In other instances, however, dose reduction remains an option, and yet another management option consists of continuing the offending drug, albeit with increased vigilance. The latter scenario is perhaps most relevant in the case of mild VAE or when disease biology dictates drug selection (i.e. T315I mutations and ponatinib treatment). Dose reduction may be of benefit in patients treated with ponatinib in particular, since a risk reduction for arterial thrombotic events of up to 40 % may be achieved by a dose reduction of 15 mg (i.e. 30 mg daily instead of 45 mg once daily) in the average dose intensity of this drug [63]. Similarly, the optimal balance between efficacy and toxicity seems to be at nilotinib 300 mg twice daily as opposed to the initial full dose of 400 mg twice daily.

In general, management of a patient with a TKI associated VAE needs to consider several factors:

- *Patient-related factors*: This includes parameters such as pre-existing vascular disease or significant cardiovascular risk factors. The susceptibility of the patient to adverse events other than VAE should also play a role in treatment decision making.
- *VAE-related factors*: Such as time elapsed from drug start to VAE onset and VAE severity.
- *Disease-related factors*: This includes parameters such as the depth of the current disease response to the TKI, as outlined in the 2013 ELN guidelines [3], as well as response duration, T315I mutation status, etc. Of note, Patients with prior vascular disease, such as PAOD, who also carry the T315I mutation, may have little choice other than ponatinib to control their CML, despite their increased risk for VAE. The current line of therapy (1^st^, 2^nd^ or more) and previous responses to other TKIs, including previous treatment failures and toxicities, should also be taken into account.

Since there are many parameters involved, decisions need to be made on a case to case basis and by a multidisciplinary team. Table 2 Summarizes the management and screening strategies for tyrosine kinase inhibitor - associated vascular adverse events.

**Table 2:** Management and screening strategies for tyrosine kinase inhibitor - associated vascular adverse events

Strategy	Comments
1. Prevention and risk assessment	
Cardiovascular risk scoring for VAE risk stratification	a) For example, the European Society of Cardiology (ESC) 2012 classification b) Reliability of such a stratification in guiding TKI drug choice is uncertain
Atherosclerosis risk factor monitoring and management (hypertension, diabetes mellitus, dyslipidemia, smoking etc.)	a) Use of accepted guidelines b) Especially important for nilotinib and ponatinib
Echocardiogram	Especially relevant for dasatinib as PAH screening
2. Monitoring tools	
Periodic cardiovascular risk score	
Atherosclerosis risk factor surveillance (hypertension, diabetes, dyslipidemia, smoking etc.)	
Subclinical radiological and/or laboratory markers	a) May include ABI, US Doppler of selected blood vessels, IMT measurement b) Clinical implication still investigational. c) ABI is the most commonly used screening measure in clinical practice
3. Treatment	
Specific treatment for vascular toxicity	Interdisciplinary approach (vascular surgeons, cardiologists/neurologists)
CML treatment modification options:	Factors to be considered:
a) Drug continuation with increased vigilance b) Drug discontinuation, choosing different TKI c) Dose reduction	a) Patient-related factors b) VAE-related factors c) Disease-related factors

All strategies are for all TKIs unless stated otherwise. See text for full details

*TKI* tyrosine kinase inhibitor, *VAE* vascular associated events, *PAH* pulmonary arterial hypertension, *ABI* ankle brachial index, *US* ultrasound, *IMT* intimal media thickness, *CML* chronic myeloid leukemia

## Conclusion

The continuing effort to achieve true personalized medicine in CML now faces the emerging and important challenge of TKI induced VAE. Currently, data regarding pathogenesis of these events is scarce, although careful patient-drug selection may be the best strategy to reduce potential vascular injury. Once such events occur, a careful evaluation of the particular circumstances is essential for suitable case management. Hopefully, continued research into the exact molecular pathways affected by the offending drugs, will improve VAE prevention, and perhaps facilitate development of new drugs with safer vascular safety profile.
